# Significant Serum Progesterone Variations on the Day of Final Oocyte Maturation in Stimulated IVF Cycles

**DOI:** 10.3389/fendo.2019.00806

**Published:** 2019-11-20

**Authors:** Upma Shanker, Barbara Lawrenz, Leif Bungum, Leyla Depret Bixio, Francisco Ruiz, Carol Coughlan, Human M. Fatemi

**Affiliations:** ^1^IVIRMA Middle East Clinic, Muscat, Oman; ^2^IVIRMA Middle East Clinic, Abu Dhabi, United Arab Emirates; ^3^Department of Obstetrics and Gynaecology, Women's University Hospital Tuebingen, Tübingen, Germany; ^4^IVIRMA Middle East Clinic, Dubai, United Arab Emirates

**Keywords:** progesterone variation, day of final oocyte maturation, ovarian stimulation, gonadotropins, elevated progesterone level

## Abstract

**Objective:** To evaluate intraday serum progesterone levels on the day of final oocyte maturation in women undergoing ovarian stimulation in a GnRH-antagonist protocol.

**Study design, size, and duration:** The study was done as a prospective observational study at a Private IVF centre in Muscat, Oman. 30 patients were recruited from May 2018 to March 2019.

**Patients:** Thirty patients with primary/secondary infertility and an indication for ovarian stimulation for IVF/ICSI treatment. The study was registered at the clinicaltrials.gov under the number: NCT03519776.

**Main outcome measures:** Progesterone levels at 4 time points (8 a.m., 11 a.m., 2 p.m. and 5 p.m.) on the day of final oocyte maturation.

**Results:** A total of 120 samples from 30 patients were included in this prospective study. Progesterone levels on the day of final oocyte maturation showed a significant decline over the day with the mean values at 8 a.m.:1.0 ng/ml, at 11 a.m.:0.8 ng/ml, at 2 a.m.: 0.7 ng/ml and at 5 p.m.:0.6 ng/ml. The difference between the first and the last progesterone level was 0.4 ng/ml, reflecting a 37.8% decline of the progesterone level within 9 h and there was a highly significant decrease in the progesterone levels recorded between 8 a.m. and 11 a.m., between 8 a.m. and 2 p.m., between 8 a.m. and 5 p.m. and 11 a.m. and 5 p.m. (*p* < 0.001).

**Conclusion:** The study findings have two clinically important conclusions: Firstly, progesterone levels on the day of final oocyte maturation decline significantly from the morning to the afternoon in patients, questioning the reliability of one arbitrarily taken progesterone level regarding the decision to perform a fresh embryo transfer or to cryopreserve the embryos. Secondly, declining progesterone levels 12 h after the last administration of gonadotropins support the theory that enhanced ovarian stimulation at the end of the follicular phase leads to an overload of the capacity of the enzymes metabolizing progesterone further on, therefore resulting in elevated progesterone levels in circulation.

## Introduction

The serum level of progesterone (P4) during the follicular phase and especially on the day of final oocyte maturation in stimulated IVF/ICSI cycles has been widely studied since the initial studies in 1991 (1). Evidence suggests that elevated progesterone levels during late follicular phase adversely affect implantation and pregnancy rates (2, 3). According to literature, the critical value above which an adverse impact on pregnancy rate occurs has been defined at 1.5 ng/ml (2). Hence, serum progesterone values during the late follicular phase of ovarian stimulation attract significant interest and the etiology of the progesterone elevation is a matter of ongoing research.

Despite the common belief that progesterone is present only during the luteal phase, a small increase in P4 levels is observed prior to the LH surge. In contrast to a natural cycle with the development of typically a single dominant follicle, the aim of ovarian stimulation for IVF is to maximize the chances of pregnancy by recruiting an adequate number of oocytes. In order to achieve this goal and following the FSH threshold concept (4), ovarian stimulation prior to IVF requires the administration of relatively high doses of exogenous gonadotropins to support multi-follicular growth (5). To prevent serum gonadotropin levels from dropping below the threshold with the consequence of follicular growth arrest, daily gonadotropin injections are required to maintain continuous stimulation of the follicles until final oocyte maturation. This results in supraphysiological serum steroid levels creating a completely different endocrine environment as compared to a natural cycle and progesterone elevation at the end of the follicular phase is documented in up to 30% of the stimulated ART cycles (6, 7).

The progesterone level on the day of final oocyte maturation is a crucial parameter regarding the decision whether to perform a fresh embryo transfer or cryopreserve the embryos. Whereas, in a natural cycle, mean levels of progesterone decline rapidly during the daytime and increase again in the early morning (8, 9), there is a lack of knowledge regarding the secretory-pattern of progesterone during the day of final oocyte maturation in stimulated cycles.

A recently published study (10) found a significant decline of progesterone levels on the day of trigger in a population of young oocyte donors, undergoing ovarian stimulation for oocyte retrieval. Contrary to a population of oocyte donors, in the “real life scenario” of an infertility clinic, the progesterone level on the day of final oocyte maturation is of utmost importance for patients who are planned to undergo a fresh embryo transfer. Therefore, this study was designed to analyse the variation of progesterone levels on the day of final oocyte maturation by measuring progesterone at four different times in a “real” infertile population.

## Materials and Methods

### Study Design and Participants

This prospective observational study was conducted from May 2018 to March 2019 in IVIRMA Fertility Clinic Muscat, Oman. Patients with primary/secondary infertility and an indication for IVF/ICSI treatment were included, fulfilling the following inclusion criteria: age 18–40 years, BMI 18–35 kg/m^2^, menstrual cycle length 24–35 days and ovarian stimulation using recombinant Follicle-stimulating-hormone (rec FSH) or human-Menopausal-Gonadotropin (hMG) in an antagonist cycle. At the time of drafting of protocol there was no published data on the fluctuation of the progesterone levels during the day of final oocyte maturation, therefore 30 patients were chosen as this trial was launched as a pilot study. All recruited patients provided written informed consent in accordance with the Declaration of Helsinki.

### Evaluation of Ovarian Reserve Parameter

The value of AMH for all patients was measured on day 2/3 of the menstrual cycle using Cobas e 411 analyzer (Roche Diagnostics, Mannheim, Germany). The measuring range is 0.01–23 ng/ml (0.07–164 pmol/L). Values below the limit of detection are reported as <0.01 ngm/ml, values above the measuring range are reported as >23 ng/ml or up to 46 ng/ml for a two-fold diluted sample. The antral follicle count (AFC) was evaluated on day 2/3 of the cycle at the time of start of ovarian stimulation. Transvaginal scans were performed using a Voluson 6 (GE Healthcare, Milwaukee, WI, USA) ultrasound machine, equipped with a 7–10 MHz two-dimensional transvaginal probe. The patients were asked to empty their bladders and were placed in the lithotomy position. For the determination of the AFC, all follicles in a size from 2 to 10 mm were counted for each ovary and summed up for the AFC result.

The samples of estrogen, progesterone, FSH, LH, AMH were collected on the first day of stimulation. The patients were followed up and reevaluated for hormones on day 8/9 of stimulation and thereafter as per individual response to treatment.

### Ovarian Stimulation and Oocyte Pick-Up Procedure

Ovarian stimulation was performed in a flexible antagonist protocol, using either rec FSH or HMG as stimulation medication and GnRH antagonist Cetrotide 0.25 {European marketing division-Serono, Mfg. date 03/2018}, or Ganirelix Acetate 0.25 mg {Organon, Mfg. date 27/10/2017} for pituitary suppression. Stimulation medication dose was individualized according to the ovarian reserve parameters (11). A daily dose of 0.25 mg of GnRH antagonist was started depending on follicular size when a dominant follicle was 14 mm in diameter. All patients were administered the gonadotropins for ovarian stimulation at 8 p.m. and GnRH antagonist for pituitary suppression at 8 a.m. If indicated, dose adjustment during ovarian stimulation was individualized according to patient's response. In case a starting progesterone elevation, in combination with a good ovarian response was seen during ovarian stimulation, gonadotropin dosage was reduced as described previously (12). As soon as three or more leading follicles ≥17 mm in diameter were observed, depending on the ovarian response, final oocyte maturation was induced by administration of either hCG (2,500, 5,000 or 10,000 IU), GnRH agonist (Triptorelin 0.3 mg) in case of risk of OHSS (13), or dual trigger (hCG and GnRH-analog).

Oocyte retrieval was carried out 36 h after administration of medication for final oocyte maturation. Two experienced reproductive medicine specialists performed the oocyte retrieval and a common technique was established for these procedures.

### Blood Collection and Hormone Measurements

The samples of FSH and LH were collected at the time of starting stimulation, which was day 2/3 of patient's menstrual cycle. They were measured by Cobas elecys 2010, modular analytics E170. The detection limits are p 0.1–200 mIU/ml (defined by the lower detection limit and the maximum of the master curve). Values below the detection limit are reported as <0.100 mIU/ml. Values above the measuring range are reported as >200 mIU/ml. Blood samples on the day of final oocyte maturation were drawn from the ante cubital vein by direct puncture. Blood sampling was performed four times during the day at an interval of 3 h: 8 a.m.,11 a.m.,2 p.m., and 5 p.m. before administration of the trigger medicine. The blood samples with a volume of 10 ml were centrifuged for 10 min at 4,000 rpm (revolution per minute) and the supernatant was retrieved and frozen at −21°C. For progesterone measurement, the assay ELECSYS® progesterone generation III was used. The measuring range is 0.159–191 nmol/L or 0.05–60 ng/ml. For detection of analytical specificity, cross-reactivities toward other hormones were used with a maximum cross-reactivity of 3.9% toward 11-Deoxycorticosterone and the minimum cross-reactivity of 0.001% toward Danazol^1^. All samples were thawed at one time and analyzed using the same assay in order to avoid any batch-to-batch variability.

### Statistical Analysis

Continuous data are summarized according to mean, standard deviation, minimum and maximum. Confidence intervals at 95% are presented for the mean values (95% CI). Progesterone levels are represented as a box plot to describe the variable distribution according to time measurements. The absolute difference between values in P4 based on pair difference for the four measurements were calculated to test mean differences. Since measures are evaluated in a single patient four times but compared two by two a dependent paired *T*-test was used to test for mean differences. Pearson's correlation coefficient (ρ) was calculated to find the correlation between the continuous parameters and the progesterone levels at the different time points. GLM(General Linear Model) procedure was applied to evaluate the relationship of categorical variables and progesterone levels at different time points.

A *p* < 0.05 is statistically significant. All analyses were performed using SAS studio (SAS® Studio).

## Results

A total of 120 samples from 30 patients were included in this prospective study. The mean age of the patients was 32.9 years (CI 95% 31.2–34.6; *SD* 4.5) and the mean BMI was 26.7 kg/m^2^ (CI 95% 25.1–28.3; *SD* 4.3). The ovarian reserve parameters showed a mean value of AMH of 3.1 ng/ml (CI 95% 2.4–3.9; *SD* 1.9) and for AFC a mean value of 18.2 (CI 95%14.9–21.5; *SD* 8.8). Out of the 30 patients included in the study 15 (50%) were cases of male factor infertility, 7 (23.3%) with ovulation disorders, 5 (16.7%) with low ovarian reserve according to Bologna criteria (14), 1 (3.3%) with endometriosis and 2 (6.7%) with infertility of unknown etiology. The baseline investigations are summarized in Table 1.

**Table 1:** Baseline parameters of the patients.

**Parameter**	**No. of patients**	**Mean ±*SD***	**95% CI**	**Min**	**Max**
Female age (years)	30	32.9 ± 4.5	31.2–34.6	23	40.0
Female BMI (kg/m^2^)	30	26.7 ± 4.3	25.1–28.3	17.96	33.8
Previous pregnancies	30	0.4 ± 0.8	0.1–0.7	0	3.0
Previous deliveries	30	0.3 ± 0.5	0.1–0.5	0	3.0
FSH on day 2/3 of cycle (IU)	30	7.3 ± 2.6	6.3–8.2	2.3	15.9
LH on day 2/3 of cycle (IU)	30	6.2 ± 2.6	5.2–7.1	1.8	11.7
AMH on day 2/3 (ng/ml)	30	3.1 ± 1.9	2.4–3.9	0.6	8.2
AFC total on day 2/3	30	18.2 ± 8.8	14.9–21.5	4.0	32.0

*AFC, antral follicle count; AMH, anti-mullerian hormone; FSH, follicle stimulating hormone; LH, luteinising hormone; BMI, body mass index*.

Ovarian stimulation for IVF/ICSI was performed in a GnRH-antagonist protocol in all patients. Stimulation length ranged from 8 to 15 days with a mean of 11.3 days (CI 95%10.7–11.8; *SD* 1.6). The average value of progesterone for reducing the dose of gonadotropin was 0.6 ng/ml (CI 95% 0.5–0.8; *SD* 0.3). On the day of final oocyte maturation, a mean follicle number of 17.8 (CI 95% 14.8–20.8; *SD* 8.0) was seen during the vaginal ultrasound. By means of oocyte pick up (OPU) procedure, a mean of number 15.1 (mean CI 95% 12.5–17.6; *SD* 6.9) were retrieved, out of which 10.7 (mean CI95%, range 8.8–12.5; *SD* 4.9) oocytes were mature(MII).The stimulation characteristics are charted in Table 2.

**Table 2:** Stimulation characteristics.

**Parameter**	**No. of patients**	**Mean ±*SD***	**95% CI**	**Min**	**Max**
Stimulation period (in days)	30	11.3 ± 1.6	10.7–11.8	8.0	15.0
Endometrial thickness on trigger day (mm)	30	7.2 ± 1.9	6.5–7.9	5.0	14.0
No of follicles (>11mm) on the day of final oocyte maturation	30	17.8 ± 8.0	14.8–20.8	4.0	30.0
Total dosage of Gn (IU)	30	3135.1 ± 1314.9	2644.1–3626.1	1500	5775
Total dosage of antagonist (mg)	30	1.5 ± 0.4	1.4–1.7	0.8	2.2
No of COCs retrieved	30	15.1 ± 6.9	12.5–17.6	2	29
No of mature oocytes	30	10.7 ± 4.9	8.8–12.5	2	22

*Gn, gonadotropins; COC, Cumulus oophorus complex; CI, confidence interval*.

**Figure 1:**
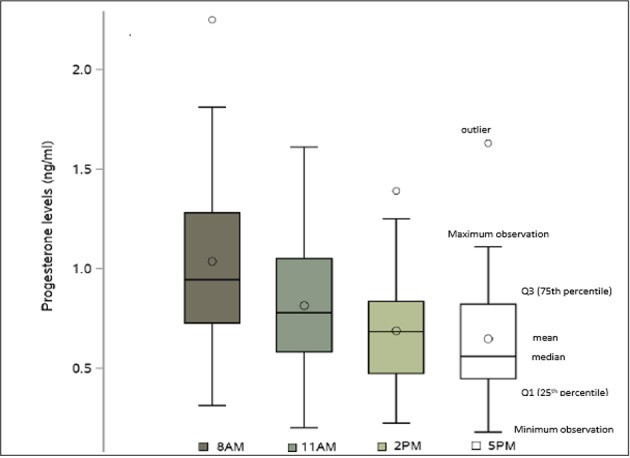
Mean and Median progesterone levels (ng/ml) on the day of final oocyte maturation.

Serum progesterone levels on the day of final oocyte maturation were highest in the morning and declined during the day, with the following mean values: at 8 a.m.:1.0 ng/ml, at 11 a.m.:0.8 ng/ml, at 2 p.m.:0.7 ng/ml and at 5 p.m.:0.6 ng/ml. The coefficient of variation for those values were 44.7, 43.8, 43.0, and 50.1 respectively to each point time. The difference between the first and the last progesterone level was 0.4 ng/ml, reflecting a 37.8% decline of the progesterone level within 9 h. The difference noted in the progesterone levels between 8 a.m. and 11 a.m., 8 a.m. and 2 p.m., 8 a.m. and 5 p.m., 11 a.m. and 5 p.m. showed a highly significant decrease (*p* < 0.0001) and a significant decrease from 11 a.m. to 2 p.m. (*p* = 0.0002). However, no significant decrease was observed between 2 and 5 p.m.The SD, 95% CI for the progesterone measurements at the different time points are given in Table 3 and the decline in progesterone values over the day is charted in Figure 1.

**Table 3:** Relationship between the different parameters and the mean progesterone levels at 8 a.m. and 5 p.m.

**Variable**	**Mean progesterone level at 8 a.m. (1.03 ng/ml)**	**Mean progesterone level at 5 p.m. (0.64 ng/ml)**
Number of follicles	*p* = 0.02	*p* = 0.21
Number of COCs retrieved	*p* = 0.11	*p* = 0.42
Number of MII	*p* = 0.03	*p* = 0.02
Type of trigger	*p* = 0.22	*p* = 0.43

*p = ANOVA. COC, Cumulus oophorus complex; MII, Mature oocytes*.

Highly significant correlations were found between the AFC and the progesterone level at 8 a.m. (*p* < 0.001) as well as between the AMH level and progesterone level at 8 a.m. (*p* < 0.001).

**Figure 2:**
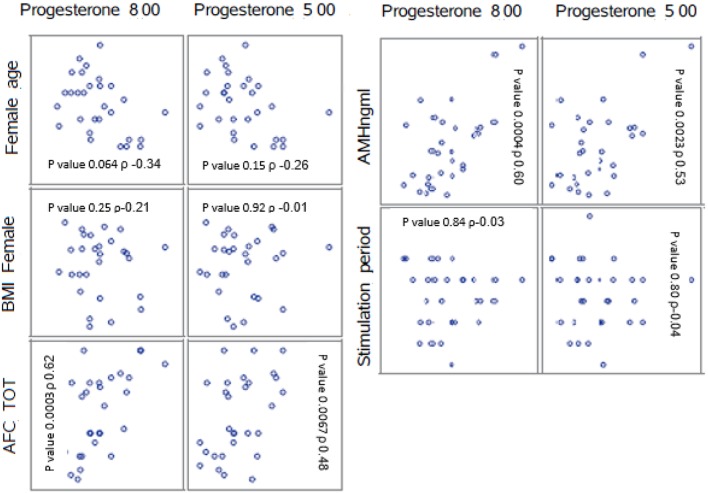
Correlations between the parameters AFC, BMI, female age, stimulation period, AMH, and the mean progesterone levels at 8 a.m. and 5 p.m. ρ = Pearson correlation coefficient. AFC, antral follicle count; BMI, body mass index; AMH, anti-mullerian hormone.

**Figure 3:**
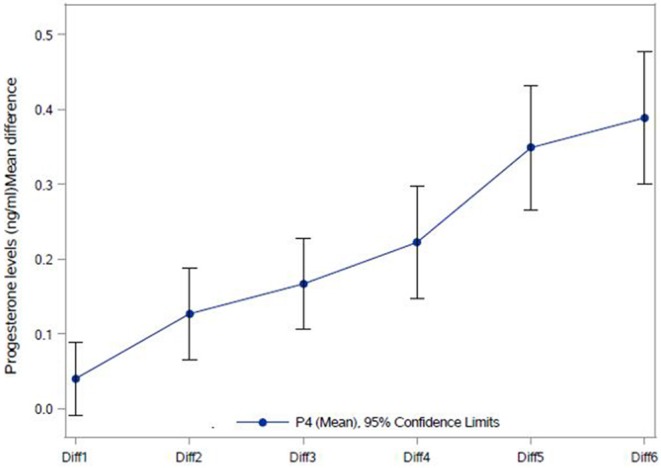
Absolute Mean difference in progesterone levels (ng/ml) on the day of final oocyte maturation. Diff1 = P4 2 p.m. – P4 5 p.m.; Diff2 = P4 11 a.m. – P4 5 p.m.; Diff3 = P4 11 a.m. – P4 2 p.m.; Diff4 = P4 8 a.m. – P4 11 a.m.; Diff5 = P4 8 a.m. – P4 2 p.m.; Diff6 = P4 8 a.m. – P4 5PM; NS, not significant. ***p < 0.0001; **p-value 0.0002.

The correlation between number of follicles seen on the day of final oocyte maturation and progesterone level at 8 a.m. was significant whereas no correlation was found to the progesterone level at 5 p.m. The values of progesterone at 8 a.m. and 5 p.m. on day of trigger did not show any significant correlation with BMI, age, values of LH, and FSH on day 2/3 of the cycle, the dose of gonadotropin or GnRH-antagonist, the number of stimulation days or type of medication for ovulation induction used. The correlations between the different parameters and the progesterone levels at 8 a.m. and 5 p.m. are summarized in Figure 2. The absolute mean difference in progesterone levels on day of final oocyte maturation is charted in Figure 3.

## Discussion

The progesterone level on the day of final oocyte maturation plays a crucial role in the decision to perform either a fresh embryo transfer or, in the event of progesterone elevation, to vitrify the embryos and perform cycle segmentation. However, the published and commonly accepted cut-off value of 1.5 ng/ml (2) is not only based on different assays used for progesterone measurement, which may impact the progesterone result (12), furthermore, it is also based on a progesterone level, taken on an arbitrary time on the trigger day. Recently, the group of González-Foruria et al. (10) published a 44.0% decline of progesterone levels over the day of final oocyte maturation in a group of 20 oocyte donors. In the herein presented study, we evaluated the course of progesterone levels on the day of final oocyte maturation in a group of 30 patients with primary/secondary infertility and an indication for ovarian stimulation for IVF/ICSI treatment. The findings of our study confirmed the findings of González-Foruria et al. (10) demonstrating a significant decline of the P4 level from the morning to the afternoon in patients who are stimulated in a GnRH-antagonist protocol, taking the stimulation medication in the evening, 12 h prior to the first P4 measurement. As afore mentioned, the event of P4 elevation on day of final oocyte maturation occurs in up to 30.0% of the stimulated ART cycles (6, 7). Elevated progesterone levels adversely affect endometrial receptivity and pregnancy rates through endometrial advancement, leading to asynchrony between endometrial development and embryo stage (15). Moreover, as per latest studies, elevated progesterone values on trigger day can negatively impact oocyte and embryo quality (16–18).

The current study, as well as the study of González-Foruria et al. (10), demonstrate clearly significant variations in daily progesterone levels on the day of final oocyte maturation. These findings question the accuracy of the single result and raise doubts, whether the reproductive medicine specialist is able to draw solid conclusions when depending on this blood test result to decide on a possible adverse impact on endometrial receptivity and embryo quality.

The clear decline of the progesterone levels over the day of final oocyte maturation, which was shown in the blood tests 12, 15, 18, and 21 h after the last gonadotropin administration in both studies confirm the theory, that elevated progesterone levels are a result of enhanced stimulation pressure on the follicles toward the end of stimulation (19, 20). Compared to our data, the study of González-Foruria (10) found a more pronounced decrease of progesterone levels over the day of final oocyte maturation (37.8 vs. 44%), most probably due to a longer duration between the first and the last progesterone measurement (9 vs. 12 h). Ovarian stimulation for IVF requires daily administration of exogenous gonadotropins to result in multifollicular growth and each of these follicles contribute to the amount of progesterone in circulation (21). The significant correlation in our study between the number of follicles seen on the day of final oocyte maturation and the progesterone level at 8 a.m. confirm the findings of Kyrou et al. (21). Furthermore, the highly significant correlation between AMH / AFC and the progesterone level at 8 a.m. underlines the risk of patients with a good ovarian reserve to develop elevated progesterone levels on the day of final oocyte maturation.

Estradiol and progesterone are products of the steroidogenesis and the concentrations of both hormones increase with growing follicle diameter (22). The enzyme CYP17 metabolizes progesterone further on, however due to low lyase activity progesterone concentration increases particularly toward the end of ovarian stimulation, causing elevated progesterone levels in stimulated IVF cycles (23). Reduction of the FSH stimulation dosage will prevent an overload of the capacity of the CYP17 enzyme activity which has been corroborated by our study, finding of a significant decline in serial progesterone levels in parallel with the subsiding effect of the stimulation medication.

The inclusion of 30 “real life” infertility patients, the use of different exogenous gonadotropins for ovarian stimulation and the measurement of all progesterone values with the same reagents, starting 12 h after the last gonadotropin injection, are the strengths of our study. A limitation may be seen in the restriction of the study to patients, being stimulated in a GnRH-antagonist protocol, as well as in the administration scheme with the gonadotropin injections taken in the evening. Therefore, these findings may not apply to the course of progesterone levels on the day of final oocyte maturation in other stimulation protocols and with a different timing of the gonadotropin injections. Further on, progesterone levels were not correlated with the reproductive outcomes as the number of participants were only 30 and due to the fact that a “freeze-all” strategy for all cycles was applied. Based on our previous publications on the etiology of progesterone serum levels during ovarian stimulation for ART (12, 19) and the knowledge gained from the current study, it maybe speculated that the diurnal serum progesterone levels would be opposite of the current findings (higher in the morning and lower in the evening), if gonadotropins would be administered in the morning. Future studies with different timing of gonadotropin administration should be performed to evaluate and possibly confirm this theory.

## Conclusions

The herein described findings of our study have an important impact on the daily work of a reproductive medicine specialist. The findings of a significant decline in progesterone levels from 12 to 21 h after the evening gonadotropin administration, on the day of final oocyte maturation underlines firstly the importance of a critical evaluation of the progesterone measurement, as this progesterone level is a crucial parameter which has a significant impact on the decision for or against a fresh embryo transfer or the need to vitrify embryos. This finding is important as it will raise the awareness of the reproductive medicine specialist that there is a fluctuation of the progesterone levels over the day of the final oocyte maturation and that in the herein described setting with the administration of the gonadotropin in the evening, progesterone levels will decline over the day. Therefore, performing the blood sample for progesterone measurement in the evening will result in a lower progesterone level compared to when the blood sample would have been taken in the morning. Secondly, it confirms the theory of enhanced stimulation pressure on the follicle being a cause of progesterone elevation and the possibility to avoid this event by reducing the stimulation dosage.

## Data Availability Statement

All datasets generated for this study are included in the article/supplementary material.

## Ethics Statement

This study was approved by the ethics committee of IVIRMA Middle East Fertility Clinic, Abu Dhabi, UAE (REFA016/2018) and the Ministry of Health, Oman. This study was registered at the clinicaltrials.gov under the number: NCT03519776. The patients/participants provided their written informed consent to participate in this study.

## Author Contributions

US substantial contribution to concept, design, acquisition, analysis, interpretation of data, manuscript preparation. BL substantial contribution to concept, design, analysis and interpretation of data, manuscript preparation, and critical revision of the article and final approval. LB contribution to concept and design of study, critical revision of the article. LD contribution to SAS programming, analysis, and interpretation of data. FR contribution to acquisition of data. CC contribution to linguistic revision of study. HF contribution to critical revision of data and final approval.

### Conflict of Interest

The authors declare that the research was conducted in the absence of any commercial or financial relationships that could be construed as a potential conflict of interest.

## Footnotes

^1^https://pimeservices.roche.com/eLD_SF/ae/en/Documents/GetDocument?documentId=4db8c0f1-4751-e711-acb2-00215a9b3428;S2 file.Product information ROCHE Cobas Progesterone III.

## References

[1] Schoolcraft W Sinton ETS Huynh D Hamilton F Meldrum DR. Lower pregnancy rate with premature luteinization during pituitary suppression with leuprolide acetate. Fertil Steril. (1999) 55:563–6. 10.1016/S0015-0282(16)54186-7 1900481

[2] Venetis CA Kolibianakis EM Bousdou JK Tarlatzis BC Progesterone elevation and probability of pregnancy after IVF; a systematic review and meta-analysis of over 60000 cycles. Hum Rep Update. (2013) 19:433–57. 10.1093/humupd/dmt014 23827986

[3] Lawrenz B Fatemi HM. Effect of progesterone elevation in follicular phase of IVF-cycles on the endometrial receptivity. Reprod Bio Med Online. (2017) 34:422–8. 10.1016/j.rbmo.2017.01.011 28162937

[4] Fauser BCJM Van Heusden AM Manipulation of human ovarian function: physiological concepts and clinical consequences. Endocr Rev. (1997) 17:121–55. 10.1210/edrv.18.1.0290 9034787

[5] Macklon NS Stouffer RL Giudice LC Fauser BC. The science behind 25 years of ovarian stimulation for *in vitro* fertilization. Endocr Rev. (2006) 27:170–207. 10.1210/er.2005-0015 16434510

[6] Ubaldi F Albano C Peukert M Riethmuller-Winzen H Camus M Smitz J Subtle progesterone rise after the administration of the gonadotropin-releasing hormone antagonist cetrorelix in intracytoplasmic sperm injection cycles. Hum Reprod. (1996) 11:1405–7. 8671476 10.1093/oxfordjournals.humrep.a019409

[7] Bosch E Valencia I Escudero E Crespo J Simon C Remohi J. Premature luteinization during gonadotropin-releasing hormone antagonist cycles and its relationship with *in vitro* fertilization outcome. Fertil Steril. (2003) 80:1444–9. 10.1016/j.fertnstert.2003.07.002 14667881

[8] Bungum L Jacobsson AK Rosen F Becker C Yding Andersen C Guner N. Circadian variation in concentration of anti-Mullerian hormone in regularly menstruating females: relation to age, gonadotrophin and sex steroid levels. Hum Reprod. (2011) 26:678–84. 10.1093/humep/deq380 21227943

[9] Bungum L Franssohn F Bungum M Humaidan P Giwercman A. The cicardian variation in anti-Mullerian hormone in patients with polycystic ovary syndrome differs significantly from normally ovulating women. PLoS ONE. (2013) 8:e68223. 10.1371/journal.pone.0068223 24023708 PMC3762839

[10] González-Foruria I Rodriguez I Martinez F Rodriguez-Purata J Montoya P Rodríguez D. Clinically significant intra-day variability of serum progesterone levels during the final day of oocyte maturation: a prospective study with repeated measurements. Hum Reprod. (2019) 34:1551–8. 10.1093/humrep/dez091 31334546

[11] La Marca A Sunkara SK. Individualization of controlled ovarian stimulation in IVF using ovarian reserve markers: from theory to practice. Hum Reprod Update. (2014) 20:124–40. 10.1093/humupd/dmt037 24077980

[12] Lawrenz B Labarta E Fatemi HM Bosch E. Premature progesterone elevation: targets and rescue strategies. Fertil Steril. (2018) 109:577–82. 10.1016/j.fertnstert.2018.02.128 29653703

[13] Papanikolaou EG Pozzobon C Kolibianakis EM Camus M Tournaye H Fatemi HM. Incidence and prediction of ovarian hyperstimulation syndrome in women undergoing gonadotropin –releasing hormone antagonist *in vitro* fertilization cycles. Fertil Steril. (2006) 85:112–20. 10.1016/j.fertnstert.2005.07.1292 16412740

[14] Ferraretti AP La Marca A Fauser BC Tarlatzis B Nargund G Gianaroli L. ESHRE consensus on the definition of ‘poor response’ to ovarian stimulation for *in vitro* fertilization: the Bologna criteria. Hum Reprod. (2011) 26:1616–24. 10.1093/humrep/der092 21505041

[15] Bosch E Labarta E Crespo J Simon C Remohi J Pellicer A Circulating progesterone levels and ongoing pregnancy rates in controlled ovarian stimulation cycle for *in vitro* fertilization: analysis of over 4000 cycles'. Human Reprod. (2010) 25:2092–100. 10.1093/humrep/deq125 20539042

[16] Huang B Ren X Wu L Zhu L Xu B Li Y. Elevated Progesterone levels on day of oocyte maturation may affect top quality embryo IVF cycles. PLoS ONE. (2016) 11:e0145895. 10.1371/journal.pone.0145895 26745711 PMC4706317

[17] Racca A Santos Ribiero S De Munck N Mackens S Drakopoulos P Camus M Impact of late-follicular phase elevated serum progesterone on cumulative live birth rates: is there a deleterious effect on embryo quality? Hum Reprod. (2017) 32:643–52. 10.1093/humrep/dey031 29481670

[18] Vanni VS Vigano P Quaranta L Pagliardini L Giardina P Molgora M. Are extremely high progesterone levels still an issue in IVF? J Endocrinol Invest. (2016) 40:69–75. 10.1007/s40618-016-0531-8 27568185

[19] Lawrenz B Belligotti F Engelmann N Gates D Fatemi HM. Impact of gonadotropin type on progesterone elevation during ovarian stimulation in GnRH antagonist cycles. Hum Reprod. (2016) 31:2554–60. 10.1093/humrep/dew213 27619773

[20] Fatemi HM Griesinger G Valerie Teal V Polyzos NP Bosch E Johnson B FSH exposure between day 8 and day of hCG administration is an independent predictor of serum progesterone rise. ESHRE Abstract 2018 (2018).

[21] Kyrou D Al-Azemi M Papanikolaou EG Donoso P Tziomalos K Devroey P The relationship of premature progesterone rise with serum estradiol levels and number of follicles in GnRH antagonist/recombinant FSH-stimulated cycles. Eur J Obstet Gynecol Reprod Biol. (2012) 13:165–8. 10.1016/j.ejogrb.2012.02.025 22425288

[22] Yding Andersen C Bungum L Nyboe Andersen A Humaidan P Preovulatory progesterone concentration associates significantly to follicle number and LH concentration but not to pregnancy rate. Reprod Biomed Online. (2011) 23:187–95. 10.1016/j.rbmo.2011.04.003 21665546

[23] Oktem O Akin N Bildik G Yakin K Alper E Balaban E. FSH stimulation promotes progesterone synthesis and output from human granulosa cells without luteinization. Hum Reprod. (2017) 32:643–52. 10.1093/humrep/dex010 28158500

